# Features of Socialization in Preschool Children with Autism Spectrum Disorder

**DOI:** 10.1192/j.eurpsy.2026.11833

**Published:** 2026-07-31

**Authors:** A. Akhmetzyanova, T. Artemyeva, A. Khamitova

**Affiliations:** Department of Psychology and Pedagogy of Special Education, Kazan Federal University, Kazan, Russian Federation

## Abstract

**Introduction:**

The socialization of children with autism spectrum disorder has specific features associated with difficulties in communication and in anticipating social situations. The level of development of anticipatory abilities and socialization skills directly affects the success of adaptation in society. A low level of anticipatory skills complicates social adaptation, which necessitates a comprehensive study of the relationship between anticipation and socialization in preschool children with autism spectrum disorder.

**Objectives:**

The study of socialization in preschool children with autism spectrum disorder.

**Methods:**

The study involved 28 preschool children with autism spectrum disorder aged 5 to 7 years. The following methods were applied: *Diagnosis of Variants of Autistic Development in Preschool Children* (N. Y. Kalmykova, M. M. Libling) for the assessment of socialization, and the authors’ method *Prognostic Stories* (A. I. Akhmetzyanova, T. V. Artemyeva) for the assessment of anticipatory abilities in various spheres of interaction and communication. Data were processed using Pearson’s correlation analysis in SPSS v.22.

**Results:**

Preschool children with autism spectrum disorder were found to have a low level of communicative skills (initiative in cooperative play, independent requests for assistance, comprehension of emotionally meaningful comments) and speech function (limited vocabulary, echolalia, monotonous intonation). While preschool children with autism spectrum disorder are capable of anticipating actions in social interaction, they experience difficulties in differentiating emotions and in formulating the verbal utterances of communication participants. A large number of children were unable to provide a verbal response to interaction scenarios, most frequently making an asocial choice. Those children who were able to provide a verbal response pointed to the norms and rules of social interaction. The participants experienced difficulty in anticipating specific events arising in various situations that occur in the course of social interaction.

**Conclusions:**

The results obtained may be used in the development of corrective and developmental programs aimed at fostering social skills in preschool children with autism spectrum disorder.

**Disclosure of Interest:**

None Declared

